# DWT features performance analysis for automatic speech recognition of Urdu

**DOI:** 10.1186/2193-1801-3-204

**Published:** 2014-04-27

**Authors:** Hazrat Ali, Nasir Ahmad, Xianwei Zhou, Khalid Iqbal, Sahibzada Muhammad Ali

**Affiliations:** Machine Learning Group, Department of Computing, City University London, Northampton Square, EC1V 0HB London, UK; School of Computer and Communication Engineering, University of Science and Technology Beijing, 100083 Beijing, China; Department of Computer Systems Engineering, University of Engineering and Technology Peshawar, 25120 Peshawar, Pakistan; Department of Electrical and Computer Engineering, North Dakota State University, Fargo, ND 58108-6050 USA

**Keywords:** Automatic speech recognition, Discrete wavelet transforms, Linear discriminant analysis, Mel-frequency cepstral coefficients, Urdu isolated words recognition

## Abstract

This paper presents the work on Automatic Speech Recognition of Urdu language, using a comparative analysis for Discrete Wavelets Transform (DWT) based features and Mel Frequency Cepstral Coefficients (MFCC). These features have been extracted for one hundred isolated words of Urdu, each word uttered by ten different speakers. The words have been selected from the most frequently used words of Urdu. A variety of age and dialect has been covered by using a balanced corpus approach. After extraction of features, the classification has been achieved by using Linear Discriminant Analysis. After the classification task, the confusion matrix obtained for the DWT features has been compared with the one obtained for Mel-Frequency Cepstral Coefficients based speech recognition. The framework has been trained and tested for speech data recorded under controlled environments. The experimental results are useful in determination of the optimum features for speech recognition task.

## Introduction

The task of Automatic Speech Recognition System may vary in terms of complexity. It might be simple to perform limited vocabulary speaker dependent recognition of isolated words under controlled environment. However, it can be too complex performing recognition of large vocabulary speaker independent continuous speech under noisy conditions. A categorization of an Automation Speech Recognition (ASR), as presented by (Varile et al. 1995), has been presented in Table 1.

**Table 1:** **Typical parameters for ASR complexity**

Parameter	Range
Speaking mode	Isolated words to continuous speech
Speaking style	Read speech to spontaneous speech
Enrollment	Speaker-dependent to speaker-independent
Vocabulary	Small (20 words) to large (20,000 words)
Language model	Finite-state to context-sensitive
Perplexity	Small (10) to large (100)
SNR	High (30 dB) to low (10 dB)
Transducer	Voice-cancelling microphone to telephone

English has a very well-established set of vowels, semi-vowels, dipthongs, nasal consonants, unvoiced fricatives, voiced fricatives, voiced, and unvoiced stops. Vowels in English can be categorized as shown in Table 2. Examples of semi-vowels include /w/, /l/, /r/, and /y/. Similarly, /ay/, /aw/, /ey/, /oy/, /o/, and /ju/ are categorized to be the diphthongs. /m/, /n/, and /ng/ are the nasal consonants. Finally, /v/, /dh/, /z/, and /zh/ are the unvoiced fricatives while /v/, /dh/, /z/, and /zh/ are listed as the voiced fricatives (Farooq and Datta 2003). This short description of the linguistics based categorization shows that English and other developed languages enjoy a well deserved attention of linguistics experts and speech processing researchers, resulting in development of more robust frameworks for ASR applications.

**Table 2:** **Vowels in english**

Vowel type	Vowel	Example
	/iv/	Beet
Front vowels	/ih/	It
	/ae/	At
	/aa/	Father
Mid position	/ax/	All
	/ah/	Up
Back vowels	/ux/	Foot
	/o/	Obey

Besides the sophisticated language resource for these languages, one of the optimization tasks for the realization of a more robust ASR system has been the extraction of features which are robust against noise. Although the Mel Frequency Cepstral Coefficients (MFCC) and the Linear Predictive Coding (LPC) based features (Hachkar et al. 2011; Han et al. 2006) have been very famous for speech recognition applications, the basic approach for these features extraction has always been based upon Short Time Fourier Transform (STFT). The features extraction based on STFT has an inherited assumption that the audio signal remains stationary throughout the period of analysis. This, in fact, has a lack of compliance to the actual scenario. Furthermore, in order to guarantee the signal to be stationary, short window duration may be used resulting in high time resolution but poor frequency resolution. Similarly, if the window duration is increased, this may improve the frequency resolution but will degrade the time resolution of the representation. The fixed window size results in a fixed resolution of the time-frequency representation of the STFT. Thus, research has been directed towards the use of Wavelet Transforms for feature extraction (Tan et al. 1996; Chang et al. 1998). This has been a source of inspiration to develop a speech recognition framework for Urdu, based upon the new Discrete Wavelet Transform based features. The lack of resource has been a practical bottleneck to drive the research work on Urdu language and speech processing. As mentioned by (Hussain 2004) and (Raza et al. 2009), Urdu is mostly written without the use of diacritics as this is the common practice by the native users. This, however, results in complexity to map the letters to sound as the diacritics represent the vowels in Urdu. Similarly for research on Urdu speech recognition, lack of enough resources on standard set of phonemes, standard speech corpus and language models have been the major challenges.

This paper presents the work on the ASR of Urdu isolated words and investigate the performance of DWT features by comparing it with the results of MFCCs. Given a carefully selected corpus and experimental conditions, this work provides a stronger baseline for future research on Urdu ASR. The remainder of this paper is organized as follows; In Section ‘Related work’, a brief overview of the research work done for development of Urdu ASR resource and framework is presented. Section ‘Overall block diagram’ briefly presents an overview of a typical speech recognition framework. In Section ‘Feature extraction by discrete wavelet transform’, the DWT features extraction has been discussed in detail. The classification achieved via LDA has been presented in Section ‘Classification’. The experimental setup and the data used in the experiment has been discussed in Section ‘Experiment’ while a comparative presentation of the experimental results has been made in Section ‘Results and comparisons’. Finally, Section ‘Conclusion and future work’ concludes the paper.

## Related work

It has not been until recently that research on speech processing of Urdu has been the topic of discussion for researchers. This includes the efforts made for corpus development as well as those towards the development of Urdu ASR. Unlike other developed languages, sophisticated categorization and resources are unavailable for Urdu, however, a basic introduction can be found in (Hussain 2004; Intermediate Urdu 2012). Raza et al. ( 2009; 2010) have made significant contribution to the development of Urdu ASR. Firstly, in (Raza et al. 2009), a speech corpus has been developed for Urdu, which is context based and phonetically rich covering all the 62 phonemes. The goal is to achieve corpus, phonetically rich and not necessarily phonetically balanced. Thus phonetic cover has been achieved but phonetic balance has not been guaranteed. Phonetic cover means that the corpus covers all the phonemes of the language while phonetic balance ensures that these phonemes occur in the corpus maintaining the ratio of occurrence in the language itself (Pineda et al. 2004). Then, in (Raza et al. 2010), they have developed ASR for spontaneous speech mixed with read speech of Urdu. The CMU Sphinx Toolkit (CMU Sphinx 2012) platform has been used for training and testing purpose. The system was trained with 87 minutes of spontaneous speech data and 70 minutes of read speech data while the testing was performed using 22 minutes of spontaneous speech data non-overlapping with the training data. The resulting Word Error Rate (WER) has a range of values for different ratios of spontaneous versus read speech in the training data. For a 0:100 ratio, the WER is 58.4, but it has significantly increased with the increase in the amount of spontaneous data, reaching a value of 18.8 for a 1:1 ratio of spontaneous vs read speech data. However, the results are based on single speaker speech recognition and extensive enhancements are required to transform the system into a multi-speaker system. (Sarfraz et al. 2010a; 2010b) has also used CMU Sphinx Toolkit towards Large Vocabulary speech recognition of Urdu. The goal was to cover the everyday speech; however, the variety in Urdu accents has not been covered as the target speech is mostly limited to suburban accent spoken in offices and homes. Furthermore, the Word Error Rates are too high for multiple speaker sets. Irtza and Hussain ( 2012) has presented the possibilities of improving the word error rates by using the approach of monitoring the word error rate improvement with increasing the training data for particular phonemes. The analysis is once again, limited to single speaker speech recognition system only. (Ali et al. 2012) has presented the development of a medium vocabulary corpus for isolated words of Urdu. The corpus comprises of 250 isolated words in Urdu, uttered by 50 speakers, with a balanced contribution from native and non-native, male and female speakers of a variety of age ranging from 20 years to 50 years. The corpus also covers various accents of Urdu as speech data of speakers from a variety of origin has been included. In (Akram and Arif 2004), the Mel-Frequency Cepstral Coefficients (MFCCs) have been extracted i.e. 39 features for a single frame of 15 milliseconds, comprising of 12 MFCCs, 12 MFCC delta features, 12 MFCC delta-delta coefficients, one 0th order cepstral coefficient and two log energy coefficients. The overall recognition rate is limited to 54 percent only. The paper lacks information on the toolkit used for the development of the framework. (Ashraf et al. 2010) has used the popular Hidden Markov Models (Rabiner 1989) for ASR of small vocabulary isolated Urdu words. The recognition performance has been reported to be very good with a mean Word Error Rate of 10.66%. Amongst the three models namely context-free-grammar, the n-gram grammar and the wordlist grammar, the simplest model i.e. the wordlist grammar model has been used. This model treats each word as a single phoneme instead of breaking it into sub-units. In the review work by (Ghai and Singh 2012), it has been mentioned that Urdu has 28 consonants and 10 vowels. (Ghai and Singh 2012) has also summarized a detailed review on the various works done in the area of Urdu ASR. The above mentioned research has been helpful to establish a baseline for future research work on Urdu ASR. However, ASR performance for DWT based features has not yet been explored for Urdu. This work presents the use of DWT based features for Urdu ASR and compares the recognition performance of the framework for DWT features with the one using MFCCs. The dataset used for the training and testing of both the frameworks is the same and both the frameworks incorporate Linear Discriminant Analysis for classification purpose.

## Overall block diagram

**Figure 1:**
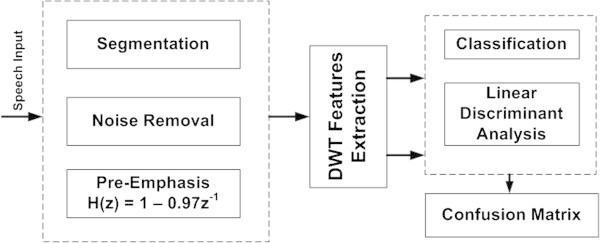
**Overall block diagram.**

The overall block diagram for a typical ASR framework is shown in Figure 1. This includes the pre-processing of the speech data, followed by the features extraction and finally the classification. The pre-processing consists of several steps. Firstly, the segmentation of the words and noise removal is achieved by using standard Adobe Audition Software. The sampling rate set throughout the processing is 16000 Hz. Isolated words are saved as.wav files in the mono format. Manual amplification or attenuation was performed wherever necessary to ensure a particular decibel level for the audio files. The next step is the pre-emphasis of the signal to enhance the energy of the higher frequency contents. The pre-emphasis of the signal is accomplished by filtering the signal, using the following equation;
1

After the noise-removal and pre-emphasis are accomplished, the input signal is provided to the feature extraction block to calculate the DWT Features.

## Feature extraction by discrete wavelet transform

### Discrete wavelet transform

The Wavelet Transform is a time-frequency transform, useful for analysis of non-stationary signals with the potential of multi-resolution. The wavelets used basis functions, are localized both in time as wells as frequency. Unlike the fixed window size used by Short Time Fourier Transform (STFT), the wavelet transform uses an adaptive window size. This means that relatively more time is allocated to the lower frequencies and less time is allocated to the higher frequencies. This kind of capability makes wavelets a promising candidate for signal and image processing (Mallat 1999). The exercise of wavelet features for speech processing is not absolutely new and has been reported by (Tan et al. 1996; Long 1999; Wassner and Chollet 1996). The selective wavelet coefficients then contribute to the feature vector. Generally, the extraction of Mel-Frequency Cepstral Coefficients incorporates the Discrete Cosine Transform, but, (Gowdy and Tufekci 2000) and (Tufekci and Gowdy 2000) have used DWT for extraction of MFCCs. A more general form of wavelet transform exists in the form of wavelet packets and has been used for speech features extraction by (Chang et al. 1998; Long and Datta 1996; 1998; Lukasia 2000). However, a major challenge arises as the wavelet packets based approaches are not robust against the shift variance, as they are usually based on the best basis selection criteria. Thus DWT based features, which are shift invariant as well as independent of speaker have been proposed by (Farooq and Datta 2003). The DWT basis function is both time localized and frequency localized with the mother wavelet or the prototype filter *ψ*(*t*), defined as given below;
2

Where, *τ* is translation parameter and *α* is a scaling parameter. *α*^-1/2^ is the energy normalization term. The mother wavelet is centered at *t* = 0, with a zero average value. For a given signal *s*(*t*), the continuous wavelet transform can be defined as;
3

**Figure 2:**
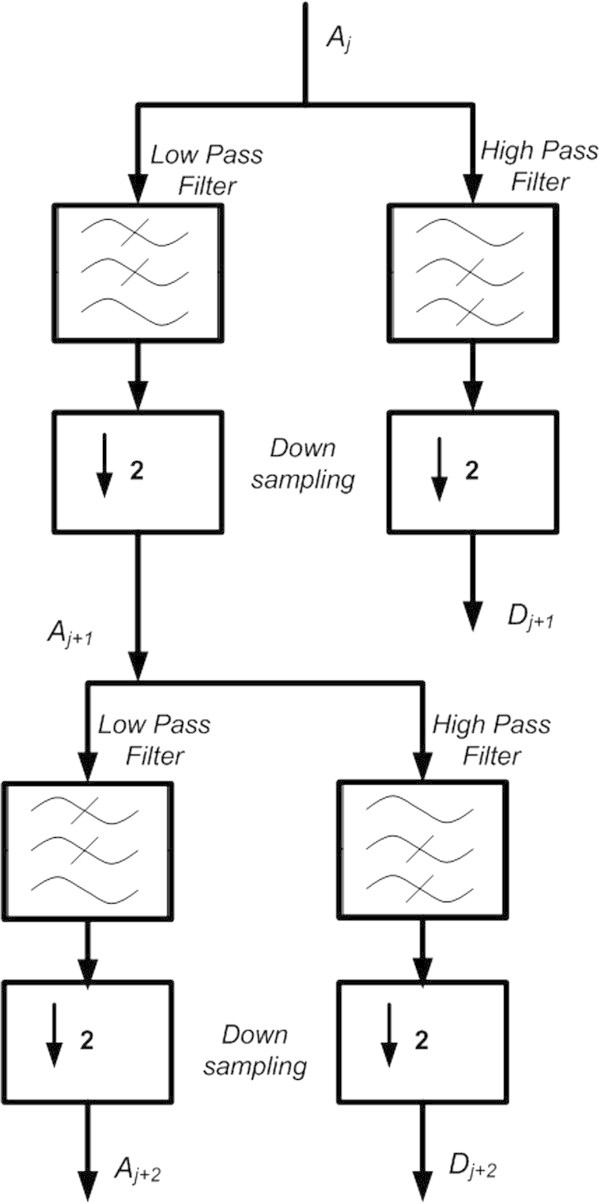
**Decomposition of Signal by DWT.**

In the above equation, *α* is the scaling parameter which gives the width of the wavelet, while the position is determined by *τ*. *ψ*^∗^(*t*) is the complex conjugate of *ψ*^∗^(*t*). The Discrete Wavelet Transform can be obtained as given below;
4

where *i*, *j* and *k* are integer values. DWT can be considered as filtering process achieved by a low pass scaling filter and a high pass wavelet filter. This transform decomposition separates the lower frequency contents and higher frequency contents of the signals. The lower frequency contents provide a sufficient approximation of the signal while the finer details of the variation are contained in the high frequency contents. In the second stage of the decomposition, the lower pass signal is further split into lower and higher frequency contents. In short, the wavelet decomposition can be referred to as a binary tree-like structure, with the left child representing the lower frequency contents, and then extension is linked to the left child, as shown in Figure 2.

### DWT features

For isolated words recognition, a primary assumption in this work is that the phoneme information has been retained after splitting a single isolated word. As a result of the DWT decomposition of the given word, the higher frequency spectral part is separated from the lower frequency spectrum. As a rule of thumb, a sampling frequency of 16 kHz has been used. A first level decomposition provides the frequency contents of 0-4 kHz and 4-8 kHz. A second level decomposition provides the frequency contents of 0-2 kHz, 2-4 kHz, and 4-8 kHz. Similarly, a third level decomposition provides the frequency contents of 0-1 kHz, 1-2 kHz, 2-4 kHz, and 4-8 kHz. Once the distribution of the speech data for a particular isolated word over different frequency bands has been accomplished, the energy for each component of the signal in the different frequency bands is determined. An essential normalization is performed on the energy values of each frequency band, by the number of samples in the respective energy band. This makes sense as the number of samples in each frequency band are not essentially uniform (Chang et al. 1998). The average energies of the different bands are the features on which the classification is based. For each single word, a total of 32 features have been obtained. These features provide the energy in each band as well as information on the temporal variation of the energy in each band.

## Classification

A supervised classification technique has been used for the word recognition task. This scenario suggests that every isolated word is a member of a pre-determined class. The classification has been achieved using Linear Discriminant Analysis (LDA) (Balakrishnama et al. 1999; Balakrishnama and Ganapathiraju 1998).

### Linear discriminant analysis

Given that {*s*[1,*i*],*s*[2,*i*],…,*s*[*n*,*i*]} to be a set of *n* examples of feature *i*, and for {*s*[1,*j*],*s*[2,*j*],…,*s*[*n*,*j*]} to be a set of *n* examples for feature *j*. Following this representation, for a pattern *k*, the features can be represented by *s*[*k*,*i*] and *s*[*k*,*j*]. For *m*[*i*] to be the mean of *i* feature, and *m*[*j*] to be the mean of *j* feature, the covariance, Σ of features *i* and feature *j* can be determined by using the following expression;
5

The Mahalanobis distance can be used in a minimum distance classifier. If *m*_1_, *m*_2_, …, *m*_*c*_ represent the means for *c* classes, and if the covariance matrices are represented by Σ_1_, Σ_2_, …, Σ_*c*_, then for the classification purpose, the Mahalanobis distance can be measured from the given feature vector to the means, and decision on the class of the given feature vector is accomplished by determining the minimum distance. The Mahalanobis distance has several advantages over Euclidean distance as it provides a correction for the different features correlation, automatically adjusts the scaling of the co-ordinate axes and is helpful in decision making process for linear as well as curved boundaries. It should be noted that shortcomings still exist in the use of Mahalanobis distance. A major challenge is posed by the quadratic rise in the required memory and processing speed resources with the increase in number of features. Furthermore, accuracy in determination of the covariance matrices cannot be guaranteed. These issues cannot prove to be devastating if the features are limited in number, however, for most of the classification task, this is not the case (Criado et al. 2011; Shen et al. 2010). The Mahalanobis distance leads to linear discriminant function when the covariance matrix Σ is the same for the data for the all the *c* classes. A general form of the Mahalanobis distance *D*, for a feature vector *v* and mean vector *m*_*v*_ and covariance matrix Σ, is given by;
6

The expression for the Mahalanobis distance can be expressed as;
7

The above expression leads to linear discriminant function if the last three terms are maximized. The linear discriminant function, *f*_*k*_(*v*) can, then, be defined as;
8

Following this reasoning, a trade-off is made for loosing decision on curved boundaries; however, memory requirements are reduced, as linear discriminant function reduces the dimensionality of the covariance matrices from *d* - *by* - *d* to *d* - *by* - 1. Besides, the computation period is also considerably reduced.

## Experiment

The experiment involved DWT features extraction for 100 isolated words of Urdu. The speech data used in this work for training and testing purpose is based upon the isolated words corpus developed by (Ali et al. 2012), which has selected the words from the list of the most frequently used words of Urdu, as listed by Center of Language Engineering (Center for Language Engineering 2012). As discussed in (Ali et al. 2012), the corpus contains a balanced distribution of data from male and female, native and non-native speakers’ of a variety of age. This framework incorporates speech data of 70% of the speakers for training purpose while testing has been achieved by using the data from the rest of the speakers. The framework ensures speaker independent recognition i.e. to eliminate inter-speaker variability. This is due to the fact that no overlap occurs between the training and test data. A sample of the representation of the speakers’ attributes has been shown in Table 3.

**Table 3:** **Representation of speaker attributes**

Speaker name	Age group	Gender	Native non-native
AAMNG1	G1	Male	Non-native
ABMNG1	G1	Male	Non-native
ACMNG2	G2	Male	Non-native
AEFYG1	G1	Female	Native
AFFYG1	G1	Female	Native
AGMNG1	G1	Male	Non-native
AHMNG1	G1	Male	Non-native

The confusion matrix determines the number of successful recognitions, as well as identifies the incorrect match confused with another word. In general, for *N* number of words, the framework will generate an *N* × *N* confusion matrix, as represented below;


For all *i* = *j*, the value of *p*_*ij*_ indicates the number of correct recognitions, while for *i* ≠ *j*, the value of *p*_*ij*_ indicates the confusion trend. For any *i*^*th*^ row, the following expression must hold true;
9

Where, *N*_*Ti*_ is the total numbers of *i*^*th*^ test words. In order to determine the accuracy rate of the framework, the fraction of the successful attempts for a particular *i*^*th*^ word can be determined by calculating the ratio of the diagonal entry to the value of *N*_*Ti*_, the total number of *i*^*th*^ test words. The fractional successful attempts, Δ_*SA*_ can be defined as;
10

Then, the percentage error for the *i*^*th*^ word can be calculated as given below.
11

## Results and comparisons

### Comparison: a word-to-word case

**Figure 3:**
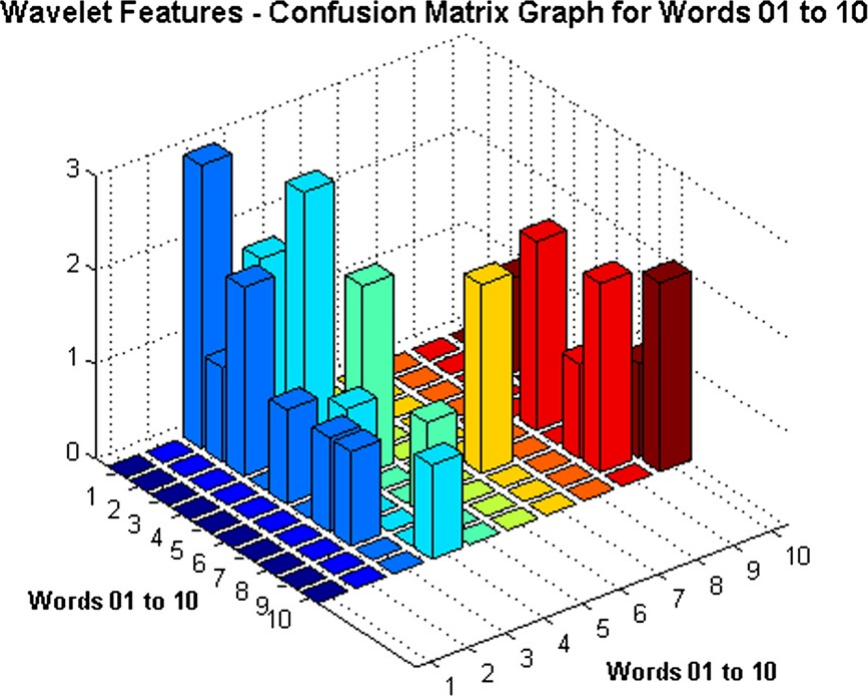
**Confusion matrix graph for words 01 to 10 - DWT features.**

**Figure 4:**
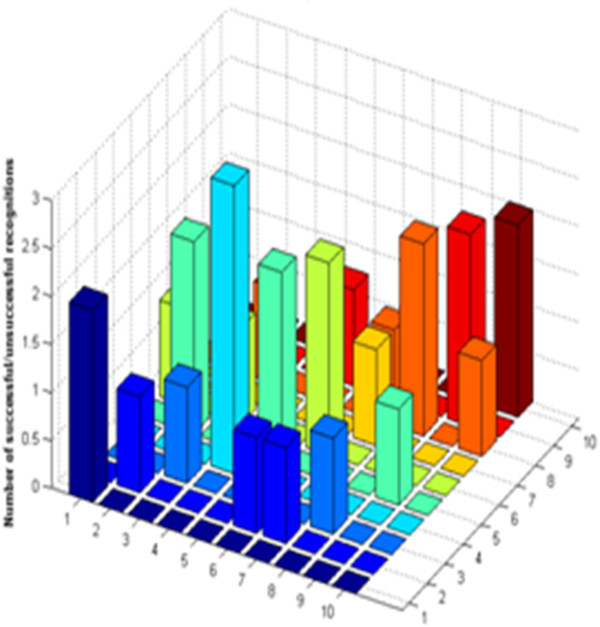
**Confusion matrix graph for words 01 to 10 - MFCC features.**

In speech recognition literature, words with extremely poor recognition are usually referred to be the bad words. However, there are some other factors that should be considered before declaration of the bad words. A poor quality of the recorded data and variations in training and testing environments are always a primary source of recognition failure. Nevertheless, in this section, the focus of discussion is the comparison of performance of DWT features with those obtained for features based on Mel-Frequency Cepstral Coefficients (MFCCs) in a recent work by (Ali et al. 2013), provided that the training and test data and the classifier used for recognition are same for both the frameworks. The comparison of the confusion matrix graph for DWT features and MFCCs clearly shows that the ratio of confused words achieved with DWT features is quite huge for DWT features. For example, the confusion matrix graph for the DWT features based ASR of the first ten words has been shown in Figure 3. For the same set of words, the confusion matrix graph for the MFCCs based ASR has been shown in Figure 4. These two results have been compared in Table 4. As shown in Table 4, the percentage error varies from 0 to 100*%*, that is for some of the words the recognition performance has been exceptional, giving 100*%* successful recognition while for some other words, the results are extremely poor with 100*%* percent error rate.

**Table 4:** **Comparison of percentage error for DWT features and MFCCs - first ten words**

Word No.	Σ _***SA***_ DWT	***%E*** DWT	Σ _***SA***_ MFCC	***%E*** MFCC
001	0	100	0.667	33.33
002	0	100	0.333	66.67
003	0.667	33.33	0.333	66.67
004	1.0	0	1.0	0
005	0.667	33.33	0.667	33.33
006	0	100	0.667	33.33
007	0.667	33.33	0.333	66.67
008	0	100	0.667	33.33
009	0.667	33.33	0.667	33.33
010	0.667	33.33	0.667	33.33

### Overall classification results comparison

**Figure 5:**
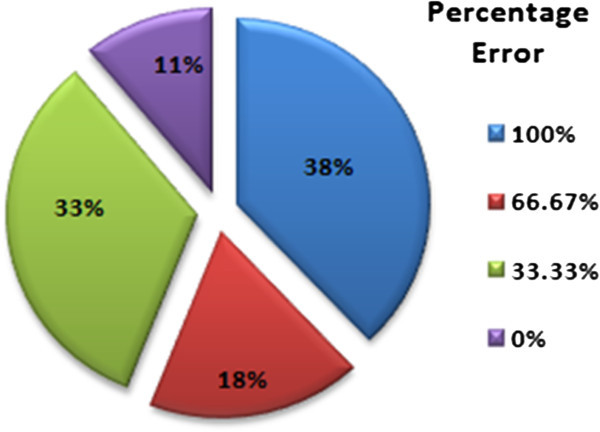
**Percentage error-wise distribution of words for DWT features based ASR.**

**Figure 6:**
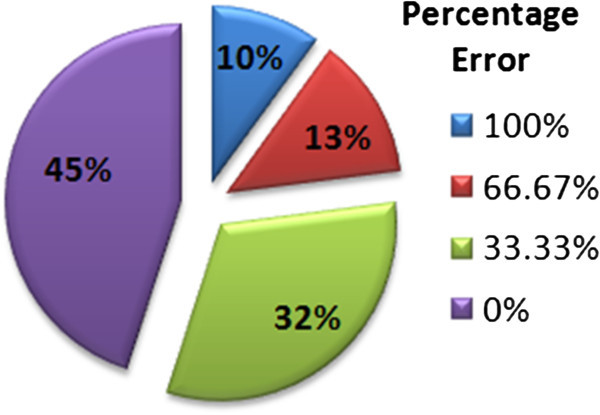
**Percentage error-wise distribution of words for MFCCs based ASR.**

Figure 5 shows the distribution of the words with respect to their percentage error for DWT features based classification. It is obvious from the distribution that the contribution of words with 100% error is quite higher i.e. 33%, as compared to the error rate for a similar data using Mel-Frequency Cepstral Coefficients, giving only 10% of test data with 100% error, as shown in Figure 6. Similarly, the words with 100% successful recognition are limited to 11%, unlike the MFCC based framework for which the 100% successful recognition contributes 45% of the graph area. The comparison of the two graphs clearly shows that the recognition rate achieved for DWT based features is far less than those achieved for MFCC based framework. The overall percentage error, *%E*, for the framework can be calculated as below;
12

Where, *α*_100_ is percentage of words with 100% error, *α*_66.67_ is the percentage of words with 66.67% error, *α*_33.33_ is the percentage of words with 33.33% error, and *α*_0_ is the percentage of words with zero error. *N*_*T*_ is the total amount of test data used. This calculation gives the value of overall error, *E* = 60.896*%*. This indeed is a very higher value as compared to *E* = 29.33*%*, achieved by using MFCCs as obvious from Table 4.

## Conclusion and future work

In this work, the ASR for a medium vocabulary of Urdu isolated words has been presented. The framework can be extended to large vocabulary applications. The ASR framework for isolated words of Urdu provides a good foundation for an extended development on continuous speech recognition framework, robust against noisy environment. The experimental results for the overall percentage error rate show that the recognition performance for DWT based features has not been promising. On the other hand, the MFFCs based classification has shown relatively better results for the same dataset. The proposed system is based on limited training data and the performance can be improved further by increasing the amount of training data. It is of key importance to mention that the results and figures presented in this work are for speech data recorded under controlled environment. Thus, a more comprehensive future task is to enhance the system and perform the training and testing for more practical speech data under noisy environments.
